# Family Support Paradox: Exploring Family Support and Life Satisfaction Among Older Adults in Rural Eastern Nepal

**DOI:** 10.3390/bs15040411

**Published:** 2025-03-24

**Authors:** Isha Karmacharya, Saruna Ghimire, Lirisha Tuladhar, Sabuj Kanti Mistry, Om Prakash Yadav, Sagar Prasai, Suresh Mehata, Uday Narayan Yadav

**Affiliations:** 1 Department of Sociology and Gerontology, Miami University, Oxford, OH 45056, USA; saruna.ghimire@miamioh.edu (S.G.); tuladhl@miamioh.edu (L.T.); 2 Scripps Gerontology Center, Miami University, Oxford, OH 45056, USA; 3 School of Population Health, University of New South Wales, Sydney, NSW 2052, Australia; smitra411@gmail.com; 4 Department of Public Health, Daffodil International University, Dhaka 1216, Bangladesh; 5 Ministry of Health, Koshi Province, Biratnagar 56613, Nepal; opyadav11120@gmail.com (O.P.Y.); sagarprasai214@gmail.com (S.P.); sureshmht@gmail.com (S.M.); 6 National Centre for Epidemiology and Population Health, The Australian National University, Canberra, ACT 2601, Australia; uday.yadav@anu.edu.au; 7 International Centre for Future Health Systems, University of New South Wales, Sydney, NSW 2052, Australia

**Keywords:** family dependency, family piety, familial support, Nepal, subjective well-being

## Abstract

This study investigated the relationship between family support (for daily activities and living) and life satisfaction among Nepali older adults, with an additional focus on gender differences. Data were drawn from a cross-sectional survey conducted in rural eastern Nepal through interviewing older adults aged 60 years and above. The total analytical sample for this study was 819. Life satisfaction was measured using Diener’s Satisfaction with Life Scale. Binary logistic regression was employed to explore the associations between reliance on family support and life satisfaction. About 57% of older adults were satisfied with their life. Older adults who relied on family support for daily activities had 51% lower odds of being satisfied with their lives compared to those who did not require such support (OR: 0.49, 95% CI: 0.35–0.69, *p* < 0.001). Gender did not moderate the relationship between family support for daily activities and life satisfaction, but interestingly, it moderated the relationship between family support for living and life satisfaction. Older men who relied on family support for living had 34% lower odds of experiencing life satisfaction compared to older women in similar situations (OR: 0.66, 95% CI: 0.48–0.90, *p* < 0.05). This study emphasizes the need for further research to comprehensively understand the impact of family support on life satisfaction among older adults in societies driven by filial piety, focusing on underlying mechanisms to confirm this paradoxical relationship.

## 1. Introduction

Life satisfaction is the self-assessment of an individual’s life based on their personal standards, which also serves as a key indicator of their overall well-being (Diener et al., 1985). It primarily focuses on the psychological dimension, highlighting subjective experiences of life contentment and fulfillment (Diener et al., 1985). Life satisfaction has been markedly used to gauge the quality of life at the individual, societal, and national levels (Veenhoven, 1996). Higher life satisfaction is positively associated with a better quality of life (Ghimire et al., 2018), increased life expectancy, and lower all-cause mortality risks (Lee & Singh, 2020). It is also widely recognized as a crucial indicator in shaping policy decisions aimed at improving psychological well-being, health behaviors, and physical outcomes (Kim et al., 2021).

Several factors influencing life satisfaction vary across different age groups due to differences in accumulated life experiences, as well as across diverse social and cultural contexts (Ji et al., 2022). Specifically, collectivist cultures, predominantly Asian cultures, prioritize group needs, social harmony, and interdependence over individual preferences (Schwartz et al., 2010). Filial piety, a core value in many collectivistic cultures, defines children’s responsibilities toward aging parents (Schwartz et al., 2010). Given Nepal’s deeply entrenched filial piety culture, as elaborated in the following paragraph, over 80% of older adults live with family members and often rely on familial support for their livelihoods (Ang & Malhotra, 2022; Singh et al., 2021). However, the extent to which older adults rely on their families may have profound implications for their autonomy, self-esteem, and overall life satisfaction and quality of life (Alonso et al., 2022; Chokkanathan & Mohanty, 2017; Heide, 2022). Thus, it becomes crucial to understand the relationship between family support and the life satisfaction of older adults within this cultural context.

Family, as a social institution, plays a central role in providing support and care to older family members in Nepal, catering to their day-to-day needs and offering various forms of assistance, including social, economic, physical, and mental support. Socially, older adults often turn to their families for companionship, emotional support, and help with basic activities of daily living (Singh et al., 2021). Pearlin and Bierman’s (2013) social stress process theory suggests that stressors from various sources—informal social circles, neighborhoods, institutions, and broader societal conditions—interact to impact well-being. It also highlights stress proliferation, where one stressor triggers others, exacerbating its effects. For instance, a serious illness in a family can lead to financial strain, shifting responsibilities, and interpersonal conflicts. Drawing upon Pearlin and Bierman’s (2013) social stress process theory, a previous study in rural India demonstrated the interplay of stressors, notably originating from familial ties, in shaping individuals’ life satisfaction (Chokkanathan & Mohanty, 2017). The study revealed that dependence on family members strains familial relationships, leading to reduced life satisfaction.

Research on life satisfaction among older adults is scarce in Nepal, particularly in rural areas. Previous studies in Nepal indicated that life satisfaction among older adults was influenced by factors such as age, marital status, education level, employment, family income, ownership of property, sufficient money for expenditure, and role in family decision-making (Ghimire et al., 2018; Karmacharya & Thapa, 2022; Shrestha et al., 2019). However, these studies were carried out in urban settings. It is well documented that significant disparities exist between urban and rural areas in Nepal across various demographic and socioeconomic dimensions (Devkota et al., 2021). Therefore, research is crucial to understanding the unique challenges and experiences of Nepal’s rural older population. Previous studies did not adequately explore the broader effects of family support within Nepal’s cultural context, nor did they thoroughly examine how this reliance affects overall life satisfaction (Ghimire et al., 2018; Karmacharya & Thapa, 2022; Shrestha et al., 2019).

Furthermore, Nepali society, marked by strong patriarchy, harbors deep-rooted gender inequality within its cultural framework (Bishwakarma, 2020). Nepali societal expectations emphasize men as providers and self-reliant individuals. Older women generally report higher satisfaction and show adeptness in family integration, including with grandchildren, sons, and daughters-in-law (Kumari & Kumar, 2023). However, previous studies provided insufficient analysis of gender differences in the relationship between family support and life satisfaction, providing opportunities for further research to elucidate these intricate dynamics.

**Figure 1:**
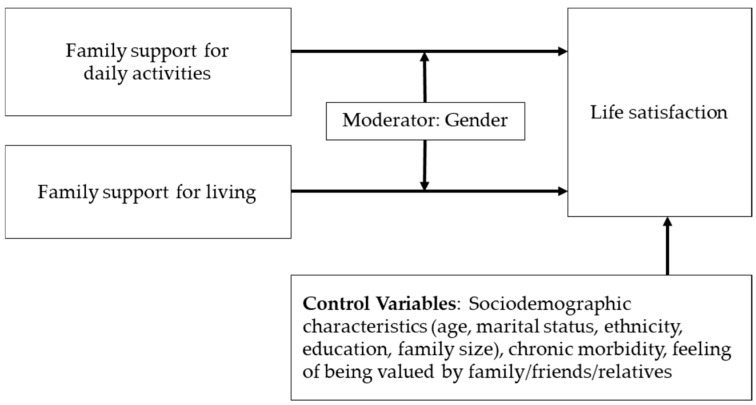
Conceptual model illustrating the relationship between family support for living and daily activities with life satisfaction among older adults in eastern Nepal.

Thus, this study aimed to examine whether reliance on family support for daily activities (e.g., bathing, dressing, personal hygiene) and living expenses (e.g., transportation, medical costs, groceries, and housing) is associated with life satisfaction among Nepali older adults. The additional aim of this study was to assess whether the relationship between family support and life satisfaction differs by gender. We hypothesized that older men who rely on family support for these needs would report lower life satisfaction compared to older women under similar circumstances. The conceptual model in Figure 1 illustrates the proposed relationships, including gender as a moderator and relevant control variables.

## 2. Materials and Methods

### 2.1. Study Design, Sample, and Sampling

This study used data from a cross-sectional study conducted between July and September 2021 in the rural settings of two districts (Sunsari and Morang) of Koshi Province in eastern Nepal. The study was conducted among older adults aged 60 years and above, as defined by Nepal’s Senior Citizens Act (Government of Nepal, 2006). The total population is 29,164,578 in Nepal, of which 2,977,318 individuals are aged 60 years and above, representing 10.2% of the total population (CBS, 2021). Sunsari and Morang are two of the most populous districts in Nepal, with total populations of 926,962 and 1,148,156, respectively (CBS, 2021). Both districts have a substantial proportion of older adults aged 60 years and above, comprising 9.5% and 10.8% of their respective populations, closely aligning with the national figure of 10.2% (CBS, 2021). The overall demographic characteristics of these districts, along with those of the province and the entire country, are provided in Supplementary Table S1. A comprehensive understanding of the study methods can be found elsewhere (Sapkota et al., 2024; Yadav et al., 2024).

The sample size was determined using a hypothesized unknown proportion of 0.5, a 5% margin of error, and a design effect of 2. To account for potential non-response, a 10% non-response rate was included. In total, 847 community-dwelling older adults participated in the survey, with a response rate exceeding 90%. Participants were recruited using a multi-stage sampling method. Two rural municipalities per district were randomly selected, followed by four wards in each municipality chosen proportionally to size. Individuals aged 60 years or older who were citizens of Nepal and had lived in the study area for at least one year were included in the study. Exclusions comprised individuals residing in nursing facilities and those with mental health conditions, hearing impairment, or communication difficulties. Additionally, for this study, individuals living alone (n = 27, 3.2%) were excluded from the study since this study was focused on familial support and its influence on life satisfaction. Family support for living had one missing value; therefore, that observation was excluded from the study. Hence, the final analytical sample included 819 respondents.

### 2.2. Data Collection

Semi-structured interviews were conducted by 12 surveyors, the majority of whom were government-employed healthcare providers in the selected districts with three-year General Medicine certificates and proficiency in local languages. Surveyors were trained via two Zoom sessions led by investigators. The training covered sessions on study tools, participant recruitment, ethics, and data collection techniques. The questionnaires used in the interviews were initially translated from English to Nepali and then verified through back-translation to ensure accuracy. The Nepali version of the questionnaire was pilot-tested among ten older adults who were not included in the final data analysis. Interviews were conducted in the Nepali language, and the data were collected using the Kobo Toolbox mobile app (KoboToolbox, 2020).

### 2.3. Variables

Life satisfaction was the dependent variable. Family support for living and daily activities were independent variables of interest. Gender (male/female) was considered as a moderator variable. Control variables included: sociodemographic characteristics, including age, marital status, ethnicity, education, family size, chronic morbidity, and a sense of being connected or valued. These variables were chosen based on prior research (Ghimire et al., 2018).

#### 2.3.1. Life Satisfaction

The 5-item satisfaction with life scale (SWLS-5), a widely used standard tool, measured respondents’ satisfaction with life (Diener et al., 1985). The SWLS-5 tool utilized in this study assessed various dimensions of an individual’s life satisfaction, including aspects such as life ideality, personal goals, and conditions. Specifically, five items in the construct stated: “In most ways, my life is close to my ideal”, “The conditions of my life are excellent”, “I am satisfied with my life”, “So far, I have gotten the important things I want in life”, and “If I could live my life over, I would change almost nothing”. Respondents were asked to rate their level of agreement with each of the five items using a 7-point Likert scale, ranging from 1 (strongly disagree) to 7 (strongly agree). To obtain a total score, the scores of the individual items were summed up, resulting in a cumulative score range of 5 to 35. The score was dichotomized as “Satisfied” (scores of 20 and above) and “Dissatisfied” (scores less than 20); following the categorization performed in previous studies (Aina et al., 2023; LaVela et al., 2019). This dichotomized life satisfaction variable was used in a binary logistic regression model.

The SWLS-5 has shown strong internal consistency, with a Cronbach’s alpha of 0.87, as well as temporal reliability with a coefficient of 0.82 (Pavot & Diener, 2009). It also exhibits good convergent validity when compared to other subjective well-being assessments (Pavot & Diener, 2009). In a previous study involving older adults aged 60 and above in Nepal, the SWLS-5 was found to have excellent reliability, with a Cronbach’s alpha of 0.93 (Ghimire et al., 2018). In this study, SWLS-5 exhibited high internal consistency with Cronbach’s alpha of 0.89.

#### 2.3.2. Family Support for Living and Daily Activities

Respondents were asked two specific questions: (1) “Do you depend on your family for living like transportation, medical expenses, groceries, and housing?” and (2) “Do you depend on your family for daily activities like bathing, dressing, personal hygiene, etc.?” Respondents answered with either “yes” or “no” to each question.

#### 2.3.3. Moderator

Gender was considered as moderator, including male and female.

#### 2.3.4. Control Variables

Sociodemographic characteristics, such as age (categorized into three groups: 60–64, 65–69, and 70+), marital status (with/without spouse), ethnicity (Dalit, Madhesi, Aadiwashi/Janajati, and Brahmin/Chettri/Thakuri), education (formal/no formal education), and family size (number of family members in the household), were included as control variables. Regarding ethnicity, there are seven main ethnic categories in Nepal: Brahmin and Chettri/Thakuri, Madhesi/other Terai castes, Dalits (untouchable caste as per traditional Hindu Caste System, and this terminology is used in a respectful way to represent this group), Newar, Aadiwashi/Janajati, Muslims, and others (Bennet et al., 2008). Historically, Brahmin and Chettri/Thakuri were considered upper-caste groups, while Dalits and Janajati were categorized as lower castes due to the entrenched hierarchical caste system, which dictated social status, occupational roles, and access to resources and opportunities (Subedi, 2016).

The sense of connection or value was also controlled in the binary logistic regression analysis. It was assessed by a question, “Do you think that you are connected to or valued by your family/friends/relatives or social networks/group you are involved in?” with a three-point Likert scale: “Most of the time”, “sometimes”, and “never feel like that”.

Considering that health status, especially chronic illnesses, has been linked to life satisfaction (Chokkanathan & Mohanty, 2017), chronic morbidity was also considered a control variable. Respondents were asked if they were ever told or diagnosed by a physician with any of the nine chronic conditions, i.e., hypertension, heart disease, stroke, lung disease (asthma, COPD, etc.), arthritis, diabetes, high cholesterol, kidney disease, and cancer. The response for each condition was reported in “Yes/No”. The number of chronic conditions was summed up and further categorized into three categories (no morbidity/single morbidity/multimorbidity).

### 2.4. Statistical Analyses

SAS version 9.4 was used for data analyses. For each variable, descriptive statistics, such as means with standard deviations and frequencies with percentages, were computed, based on their respective measurement types. For bivariate analyses, chi-square tests were conducted for categorical predictors, and independent samples *t*-tests were used for numeric predictors with respect to the binary dependent variable.

Binary logistic regression was performed to examine the relationship between predictor and dependent variables and to assess whether gender moderated these relationships. Key independent and control variables were included in the model based on theoretical relevance. Multicollinearity was assessed using the “proc reg” statement, with variance inflation factors for all variables found to be below 2.5, indicating no significant multicollinearity concerns (Johnston et al., 2018). As a result, all key variables were retained for the logistic regression model. Advanced diagnostics, including dfbetas, influence, and leverage plots generated through the “proc logistic” statement, revealed no issues with influential observations. Model predictability was assessed using concordance statistics (c-statistics), with values ranging from 0.61 to 0.80 considered good, and 0.81 to 1.00 considered very good (Kwiecien et al., 2011). Moderation by gender was assessed by including an interaction term between gender and family support for daily activities and for living in separate regression models. Statistical significance was determined using a *p*-value threshold of <0.05.

## 3. Results

### 3.1. Study Respondents’ Characteristics

The characteristics of respondents and bivariate analyses by life satisfaction are presented in Table 1. Among the total respondents (n = 819), the sample was almost evenly distributed across three age groups. More than half of the respondents were men (55.7%), the majority were married (77.9%) and had no formal education (89.3%). The highest percentage of older adults identified themselves as Madhesi ethnicity (41.5%), followed by Aadiwashi/Janajati (25.4%), Dalit (23.0%), and Brahmin/Chettri/Thakuri (10.1%). The mean household family size reported was about seven. Only one-fifth of participants reported feeling valued most of the time, while the majority felt valued only occasionally (63.1%) or never (16.9%). Nearly half (46%) reported having one or more chronic conditions. Dependency on family was also common, with 47.4% relying on family for daily activities and 79.2% dependent on family for living. Despite these challenges, more than half (57.1%) of respondents expressed satisfaction with their lives.

Bivariate analyses revealed several significant associations between various factors and life satisfaction. Among the control variables, life satisfaction varied significantly based on participants’ ethnicity, education, family size, sense of being valued, and presence of chronic conditions. Family support for daily activities was strongly associated with life satisfaction (*p* < 0.001), whereas no statistically significant relationship was observed between family support for living and life satisfaction.

### 3.2. Binary Logistic Regression for Family Support and Life Satisfaction

Results from the unadjusted and adjusted binary logistic regression models are presented in Table 2. Separate models were run for family support for daily activities (Model 1) and for living (Model 2). The predictability of both models was good, with c-values of 0.758 and 0.747 for Model 1 and Model 2, respectively. In the adjusted model, family support for daily activities was significantly associated with life satisfaction (Table 2, Model 1). Older adults who relied on family support for daily activities had 51% lower odds of being satisfied with their lives compared to those who did not require such support (OR: 0.49, 95% CI: 0.35–0.69, *p* < 0.001). In contrast, family support for living showed no significant association with life satisfaction (Table 2, Model 2).

Among the control variables, ethnicity played a key role, with Aadiwashi/Janajati and Brahmin/Chettri/Thakuri groups reporting higher life satisfaction compared to participants from the Dalit ethnic group, while the findings were statistically non-significant for the Madhesi group. Both family size and chronic morbidity, including single and multimorbidity, were associated with lower odds of life satisfaction. Additionally, a strong sense of being valued was crucial for life satisfaction, with those feeling devalued having significantly lower odds of satisfaction.

### 3.3. Family Support and Life Satisfaction: Moderation by Gender

**Figure 2:**
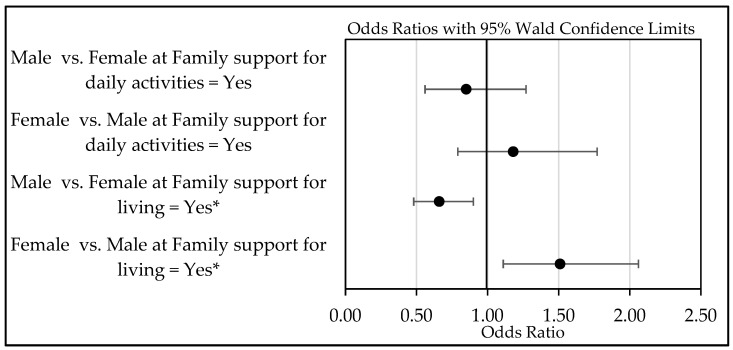
Forest plot illustrating the moderation effect of gender in relationships of life satisfaction with family support for daily activities and living. * Statistical significance with *p* < 0.05.

Figure 2 illustrates the moderation effect of gender in the relationship between life satisfaction and family support for daily activities and living. The forest plot shows no statistically significant gender difference in how family support for daily activities influences life satisfaction; i.e., for male vs. female (OR: 0.85, 95% CI: 0.56–1.27, *p* > 0.05) and female vs. male (OR: 1.18, 95% CI: 0.79–1.77, *p* > 0.05).

However, the plot reveals a notable difference in the relationship between family support for living and life satisfaction. For men, the odds of life satisfaction with family support for a living are lower than for women in similar situations. Specifically, older men who relied on family support for living had 34% lower odds of experiencing life satisfaction compared to older women in similar situations (OR: 0.66, 95% CI: 0.48–0.90, *p* < 0.05). In other ways, among older adults who relied on family support for living, women were 51% more likely to be satisfied with their lives than men (OR: 1.51, 95% CI: 1.11–2.06, *p* < 0.05).

## 4. Discussion

This study investigated the association between family support and life satisfaction of older adults in rural eastern Nepal. While relying on family support for daily activities was associated with lower odds of life satisfaction, depending on family for living did not generally impact life satisfaction—except when considering gender. Among older adults who relied on family support for living, men were less likely to feel satisfied with their lives compared to women.

We found that more than half of respondents reported being satisfied with their lives, while 42.9% expressed dissatisfaction—more than double the prevalence found in previous studies in Nepal (Ghimire et al., 2018; Shrestha et al., 2019). This discrepancy may be attributed to differences in the study settings, as the current research was conducted in a rural area, whereas the earlier studies focused on urban populations. Rural older adults may report lower life satisfaction than their urban counterparts due to limited access to healthcare and social services in rural settings, which can diminish their sense of security and overall well-being (Rey-Beiro & Martínez-Roget, 2024).

Reliance on family support for daily activities was associated with decreased odds of life satisfaction, a finding consistent with the social stress process theory (Pearlin & Bierman, 2013). This aligns with previous research indicating that poor health status can increase family strain, which in turn negatively affects subjective well-being (Chokkanathan & Mohanty, 2017). Higher dependency on family members appears to exacerbate this strain, highlighting the critical role of dependency in mediating the relationship between health status and life satisfaction among older adults (Chokkanathan & Mohanty, 2017). While traditional Nepali cultural norms rooted in filial piety emphasize the importance of family support for the well-being of older individuals (Singh et al., 2021), an overreliance on familial assistance may inadvertently reduce life satisfaction. This paradox could stem from compromised autonomy, where individuals feel a loss of independence, or from feelings of being a burden on their family (Bishwakarma, 2020; Heide, 2022). These dynamics suggest a nuanced relationship between family support and subjective well-being. However, the mechanisms underlying this association remain unclear, as exploring them was beyond the scope of this study. Future research should investigate these processes in greater depth to uncover the pathways through which family support impacts life satisfaction.

Interestingly, family support for living did not show a statistically significant association with life satisfaction, a finding that diverges from expectations. This result suggests that older adults may perceive family support for living differently from support for daily activities. Support for living, such as financial assistance or housing, may not carry the same emotional weight as assistance with daily tasks, which can challenge an individual’s sense of independence. This distinction underscores the importance of examining the context and nature of family support in influencing well-being.

Gender did not significantly moderate the association between family support for daily activities and life satisfaction among older adults, but it played a notable role in the relationship between family support for living and life satisfaction. Specifically, older men who relied on family support for living reported lower life satisfaction than older women in similar circumstances. This finding highlights how societal gender norms influence perceptions of family support. In societies with strong patriarchal norms, such as Nepal, men are traditionally viewed as providers and symbols of self-reliance (Bishwakarma, 2020). When men rely on family support for a living—whether due to retirement, job loss, or unemployment—they may experience internal conflict with societal expectations and personal beliefs about their role within the family. This disconnect can result in feelings of inadequacy, frustration, loss of independence, and ultimately lower life satisfaction (Ang & Malhotra, 2022). Conversely, women in these settings are often expected to manage household responsibilities rather than engage in financial or external work (Bishwakarma, 2020). As a result, reliance on family support for living may align with their traditional roles, leading to a more positive perception of such support. This internalized acceptance of dependency may amplify the positive impact of family support on women’s life satisfaction in later life.

These findings illustrate the paradoxical nature of family support in later life, particularly the differing perceptions between men and women. While family support is critical for many older adults, it can also challenge their autonomy and redefine their role within the family. This tension is particularly evident in discussions surrounding filial piety, where the expectation of care from children can create a delicate balance between the need for support and the desire for independence (Ang & Malhotra, 2022). The results of this study emphasize the need to consider gender dynamics when exploring the relationship between family support and life satisfaction among older adults.

A brief discussion of the control variables is also warranted, as this study highlighted several factors influencing life satisfaction among older adults, including ethnicity, family size, sense of being valued, and chronic morbidity. Specifically, Aadiwashi/Janajati and Brahmin/Chettri/Thakuri ethnic groups reported higher life satisfaction compared to Dalit participants. Conversely, larger family sizes, chronic morbidity, and a lack of feeling valued were all associated with lower odds of life satisfaction. Members of Dalit ethnic groups, historically disadvantaged and socially excluded in Nepal (Atreya et al., 2022), may experience lower life satisfaction due to systemic inequities and caste-based discrimination, which limit their access to resources and opportunities. An intriguing finding of this study was that each additional family member reduced the odds of experiencing life satisfaction by 16%. This diverges from previous research, which typically highlights family support, in terms of large family size, as a positive contributor to life satisfaction in later life (de Oliveira et al., 2019; Wang et al., 2020). A possible explanation lies in the quality of relationships within the family. While larger families may offer more potential sources of support, they can also introduce greater interpersonal conflicts, stressors, and responsibilities. According to the social stress process theory, such stressors may outweigh the benefits of social support and negatively impact well-being (Pearlin & Bierman, 2013). Moreover, the rural context of this study provides additional insights. In resource-constrained settings, larger families may stretch available resources thin, leading to reduced individual attention and support for older adults. This scarcity can foster feelings of neglect and devaluation, ultimately diminishing life satisfaction (Chokkanathan & Mohanty, 2017). Feeling consistently valued emerged as another important factor, as it can boost self-esteem, reduce stress, and foster stronger social connections, all of which contribute positively to life satisfaction (Rey-Beiro & Martínez-Roget, 2024). In contrast, chronic conditions and multimorbidity were associated with lower life satisfaction, likely due to the physical limitations, social isolation, and reduced work capacity that often accompany these conditions (Vancampfort et al., 2017). These health challenges can profoundly disrupt daily life and social relationships, further diminishing well-being.

## 5. Strengths and Limitations of the Study

One notable strength of this study was its large sample size, which, despite the challenges of the COVID-19 pandemic, ensured a robust representation of older adults with diverse gender and age characteristics. Conducting interviews in the Nepali language likely enhanced communication between interviewers and participants, fostering a better understanding of responses. Additionally, pilot testing the interview questions strengthened the study’s methodological rigor by improving the validity and reliability of the data collection process.

However, certain limitations should be noted. As a cross-sectional design, it cannot establish causal relationships between family support and life satisfaction, limiting the ability to determine how family dynamics influence well-being over time. Furthermore, the study utilized only two single items to measure family support, which may not comprehensively capture its complexity. The research was limited to rural settings, leaving unanswered questions about how family dynamics and life satisfaction might differ in urban environments. Additionally, the study did not account for the dependence on adult children or family members who had migrated temporarily for work, a significant factor given Nepal’s prevalent migration patterns among youth (Adhikari et al., 2023). Data collection during the COVID-19 pandemic presents another potential limitation. Lockdown measures may have restricted mobility, particularly for older adults, potentially inflating responses regarding reliance on family for daily activities and living (Felipe et al., 2023).

## 6. Study Implications and Future Research

The study underscores the intricate nature of family support for older adults in rural Nepal, highlighting the importance of strategies that prioritize the independence and well-being of older family members. Policy frameworks must not only endorse and reinforce the significance of familial care, particularly for older adults who need support with their daily activities, but also underscore the importance of preserving individual autonomy within familial support structures (Alonso et al., 2022; Chokkanathan & Mohanty, 2017; Heide, 2022). Encouraging intergenerational communication and understanding within families can foster an environment conducive to mutual respect and support. Moreover, implementing culturally sensitive educational programs aimed at both older adults and their families becomes imperative. These programs could emphasize the evolving needs and preferences of the older population in rural settings while empowering families with the tools to offer assistance without undermining the older individual’s independence. Additionally, initiatives that highlight the value of active participation and decision-making by older adults in familial affairs could strengthen their sense of agency and strengthen familial relationships.

Interestingly, this study reveals that men who rely on family for a living are less likely to experience life satisfaction, whereas this reliance appears to have the opposite effect on women. To gain a deeper understanding of this relationship over time, longitudinal studies could provide valuable insights into the evolving dynamics of gender, family support, and life satisfaction among older individuals. One avenue for exploration could involve examining how shifts in societal norms, economic conditions, and caregiving responsibilities influence the experiences of older women. For example, changes in women’s workforce participation, access to education and healthcare, and evolving family structures may impact their perceptions of family support and life satisfaction. In addition to longitudinal research, qualitative studies could provide deeper insights into the lived experiences of older adults. By capturing individual narratives, qualitative methods can help uncover the emotional, cultural, and contextual factors that influence how family support is perceived and its impact on life satisfaction.

Furthermore, this study also underscores the necessity of investigating the stressors experienced by caregivers and providers within families responsible for the care of older family members, especially given the absence of caregiver incentives, support policies, and programs in Nepal (Singh et al., 2021). Future studies could explicitly investigate, which forms of family support (e.g., financial, emotional assistance, healthcare support, transportation, etc.) are of major significance for older adults, focusing interventions on areas of family support that are most needed and impactful to improve their well-being. Furthermore, future research could delve deeper into the underlying mechanisms that elucidate the paradoxical nature of family support concerning the life satisfaction of older adults. This is particularly relevant within the cultural context of filial piety, where the expectation of care can both strengthen family bonds and introduce stressors that impact the life satisfaction of older adults.

## Figures and Tables

**Table 1:** Respondents’ characteristics and bivariate analyses by life satisfaction.

Variables		Life Satisfaction		*p*-Value
	Total (n = 819, 100%)	Satisfied (n = 468, 57.1%)	Dissatisfied (n = 351, 42.9%)	
	n (%)	n (%)	n (%)	
Age (in years)				0.130
60–64	280 (34.2)	172 (36.7)	108 (30.8)	
65–69	276 (33.7)	157 (33.6)	119 (33.9)	
70 and above	263 (32.1)	139 (29.7)	124 (35.3)	
Gender				0.174
Female	363 (44.3)	217 (46.4)	146 (41.6)	
Male	456 (55.7)	251 (53.6)	205 (58.4)	
Marital status				0.437
With spouse/partner	638 (77.9)	360 (76.9)	278 (79.2)	
Without spouse/partner ^#^	181 (22.1)	108 (23.1)	73 (20.8)	
Ethnicity				**<0.001**
Dalit	188 (23.0)	88 (18.8)	100 (28.5)	
Madhesi	340 (41.5)	164 (35.0)	176 (50.1)	
Aadiwashi/Janajati	208 (25.4)	161 (34.4)	47 (13.4)	
Brahmin/Chettri/Thakuri	83 (10.1)	55 (11.8)	28 (8.0)	
Education				**0.015**
Formal	88 (10.7)	61 (13.0)	27 (7.7)	
No formal education	731 (89.3)	407 (87.0)	324 (92.3)	
Family size (Mean ± SD)	6.9 ± 2.6	6.5 ± 2.7	7.6 ± 2.4	**<0.001 ^a^**
Sense of being valued				**<0.001**
Most of the time	164 (20.0)	142 (30.3)	22 (6.3)	
Sometimes	517 (63.1)	271 (57.9)	246 (70.1)	
Never	138 (16.9)	55 (11.8)	83 (23.6)	
Chronic morbidity				**<0.001**
No morbidity	446 (54.4)	296 (63.2)	150 (42.7)	
Single morbidity	229 (28.0)	108 (23.1)	121 (34.5)	
Multimorbidity	144 (17.6)	64 (13.7)	80 (22.8)	
Family Support for daily activities				**<0.001**
No	431 (52.6)	298 (63.7)	133 (37.9)	
Yes	388 (47.4)	170 (36.3)	218 (62.1)	
Family support for living				0.123
No	170 (20.8)	106 (22.6)	64 (18.2)	
Yes	649 (79.2)	362 (77.4)	287 (81.8)	

Notes: ^a^ *p*-value from independent samples *t*-test and others are from chi-square test. Significant *p*-values are bolded; ^#^ Without spouse/partner included unmarried, divorced, widow/widower.

**Table 2:** Binary logistic regression for family support and life satisfaction.

Variables	Unadjusted	Model 1 ^a^	Model 2 ^b^
	OR (95% CI)	OR (95% CI)	OR (95% CI)
Age (in years)			
60–64	Ref.	Ref.	Ref.
65–69	0.83 (0.59–1.16)	0.74 (0.50–1.10)	0.87 (0.59–1.27)
70 and above	0.70 (0.50–0.99)	0.70 (0.46–1.09)	0.80 (0.53–1.23)
Gender			
Female	Ref.	Ref.	Ref.
Male	0.82 (0.62–1.09)	0.96 (0.68–1.34)	0.96 (0.68–1.36)
Marital status			
With spouse/partner	Ref.	Ref.	Ref.
Without spouse/partner ^#^	1.14 (0.82–1.60)	1.31 (0.87–1.99)	1.25 (0.82–1.88)
Ethnicity			
Dalit	Ref.	Ref.	Ref.
Madhesi	1.06 (0.74–1.51)	0.99 (0.66–1.48)	0.96 (0.64–1.43)
Aadiwashi/Janajati	3.89 (2.52–6.00) ***	2.19 (1.34–3.58) **	2.33 (1.44–3.79) ***
Brahmin/Chettri/Thakuri	2.23 (1.30–3.82) ***	2.00 (1.06–3.77) *	2.11 (1.13–3.94) *
Education			
Formal	Ref.	Ref.	Ref.
No formal education	0.56 (0.35–0.89) *	0.80 (0.46–1.41)	0.68 (0.39–1.20)
Family size	0.84 (0.80–0.89) ***	0.92 (0.85–0.99) *	0.92 (0.85–0.98) *
Sense of being valued			
Most of the time	Ref.	Ref.	Ref.
Sometimes	0.17 (0.11–0.28) ***	0.30 (0.18–0.50) ***	0.26 (0.15–0.43) ***
Never	0.10 (0.06–0.18) ***	0.11 (0.06–0.20) ***	0.10 (0.06–0.19) ***
Chronic morbidity			
No morbidity	Ref.	Ref.	Ref.
Single morbidity	0.45 (0.33–0.63) *	0.62 (0.43–0.89) *	0.51 (0.36–0.72) ***
Multimorbidity	0.41 (0.28–0.59) **	0.50 (0.32–0.80) **	0.38 (0.24–0.59) ***
Family support for daily activities			
No	Ref.	Ref.	-
Yes	0.35 (0.26–0.46) ***	0.49 (0.35–0.69) ***	
Family support for living			
No	Ref.	-	Ref.
Yes	0.76 (0.54–1.08)		1.23 (0.80–1.90)

Notes: Ref = Reference category; OR = Odds Ratio; CI = Confidence Interval. n = 819. * *p* < 0.05; ** *p* < 0.01; *** *p* < 0.001. ^#^ Separate/divorced, widow/widower, and unmarried were combined into “without spouse/partner”. Model 1 and Model 2 are run separately for the two primary predictors of interest: family support for daily activities and family support for living, respectively. Both models were adjusted for control variables, including age, marital status, ethnicity, education, family size, a sense of being connected or valued, and chronic morbidity. ^a^ R-Square = 0.200, Max-rescaled R-Square = 0.268; c-statistic = 0.758. ^b^ R-Square = 0.184, Max-rescaled R-Square = 0.247; c-statistic = 0.747.

## Data Availability

The datasets generated and/or analyzed during the current study are not publicly available due to privacy and confidentiality concerns. The data may contain sensitive information about individuals’ demographics, and experiences, physical and mental health status, which must be protected to ensure the privacy and dignity of the participants. However, the de-identified datasets are available on reasonable request with ethical approval.

## Supplementary Materials

The following supporting information can be downloaded at: https://www.mdpi.com/article/10.3390/bs15040411/s1, Table S1: Demographic characteristics of Morang and Sunsari districts.
