# Supplementary material for: Family Support Paradox: Exploring Family Support and Life Satisfaction Among Older Adults in Rural Eastern Nepal
Source: Behav Sci (Basel). 2025 Mar 24;15(4):411. doi: 10.3390/bs15040411 (PMC12024343; doi:10.3390/bs15040411)
Supplement: Supplementary file 1 [file behavsci-15-00411-s001.zip › behavsci-3370372-supplementary.pdf]

Supplementary Table S1. Demographic characteristics of Morang and Sunsari districts

Indicators	Nepal	Koshi Province	Morang	Sunsari
Total Population	29,164,578	4,961,412	1,148,156	926,962
60+ Population	2,977,318	556,464	123,709	88,482
Avg. HH Size	4.37	4.16	4.22	4.36
Population Density	198	192	619	737
Annual Growth Rate	0.92	0.86	1.66	1.86
Sex Ratio (Males per Females)	95.59	95.02	94.39	93.95
Literacy	76.2%	79.7%	78.6%	78.1%
Aging Index	24.9%	28.9%	27.9%	23.1%
Old-age Dependency Ratio	16.5%	17.9%	17.0%	15.1%

Data Source: National Population and Housing Census 2021, Nepal.
